# Efficacy of Ceftazidime-avibactam in treating Gram-negative infections: a systematic review and meta-analysis

**DOI:** 10.1007/s10096-025-05044-5

**Published:** 2025-01-22

**Authors:** Nahal Khoshdel, Melina Noursalehigarakani, Zahra Sadat Seghatoleslami, Fahimeh Hadavand, Elaheh Eghbal, Mohammad Javad Nasiri, Elena Simula, Parnian Ahmed, Leonardo Antonio Sechi

**Affiliations:** 1 School of Medicine, Shahid Beheshti University of Medical Sciences, Tehran, Islamic Republic of Iran; 2 Department of Infectious Diseases, Islamic Azad University, Tehran Medical Branch, Tehran, Islamic Republic of Iran; 3 University of Sassari, Sassari, Italy

**Keywords:** Ceftazidime-avibactam, CAZ-AVI, Gram-negative infections, CAZ-non-susceptible pathogens, Efficacy, Systematic review, Meta-analysis, Antibiotic resistance, Clinical success, Microbiological success

## Abstract

**Introduction:**

Ceftazidime-avibactam (CAZ-AVI) has emerged as a promising treatment option for Gram-negative infections, particularly those caused by CAZ-Non-Susceptible (NS) pathogens. This systematic review and meta-analysis aim to assess the efficacy and safety of CAZ-AVI in these challenging infections.

**Methods:**

We systematically queried EMBASE, Cochrane CENTRAL, and PubMed/Medline for studies published until September 15, 2024. Randomized Controlled Trials (RCTs) evaluating CAZ-AVI against Gram-negative infections were included. A meta-analysis was performed to calculate pooled odds ratios (OR) for both clinical and microbiological success.

**Results:**

A total of 146 studies were identified through database searches, leading to the inclusion of 17 studies. Among the efficacy studies for Gram-negative pathogens, there was no significant difference in clinical success rates for CAZ-AVI compared to comparators (pooled OR: 0.90, *p* = 0.22), and a non-significant increase in microbiological success was observed (pooled OR: 1.20, *p* = 0.41). In contrast, for CAZ-NS pathogens, six studies reported no significant difference in clinical cure rates (pooled OR: 0.77, *p* = 0.24), while four studies indicated a non-significant increase in microbiological cure rates (pooled OR: 1.83, *p* < 0.02).

**Conclusions:**

This study suggests that CAZ-AVI is a viable option for treating Gram-negative infections, including CAZ-NS pathogens. While it has shown promising activity against these resistant pathogens, its clinical and microbiological success rates are comparable to other antibiotics in the overall analysis. However, CAZ-AVI may offer an advantage in managing resistant infections. These findings underscore the need to consider CAZ-AVI in treatment guidelines and emphasize the importance of antibiotic stewardship programs to optimize its use and prevent resistance. Ongoing monitoring of resistance patterns and patient outcomes is essential to ensure its long-term efficacy.

## Introduction

Ceftazidime-avibactam (CAZ-AVI) is a novel beta-lactam/beta-lactamase inhibitor combination that has gained attention for its efficacy against multidrug-resistant Gram-negative bacteria [1]. With the increasing prevalence of infections caused by resistant strains, particularly those that are non-susceptible to traditional antibiotics, there is an urgent need for effective therapeutic options [2]. CAZ-AVI has demonstrated significant activity against a range of pathogens, including *Enterobacteriaceae* and *Pseudomonas aeruginosa*, making it a valuable addition to the antimicrobial arsenal [3, 4].

However, while CAZ-AVI was primarily developed to target Class A beta-lactamases, including KPC-producing *Enterobacteriaceae*, its use must be clarified. CAZ-AVI is not typically intended for treating Gram-negative bacteria (GNB) that are susceptible to ceftazidime alone, nor is it the preferred option for ESBL- or AmpC-producing bacteria. Additionally, for infections caused by carbapenem-resistant *P. aeruginosa* that do not produce carbapenemases, ceftolozane-tazobactam (CTLZ/TAZ) has shown to be more effective. In this study, we aim to evaluate the efficacy of CAZ-AVI specifically against resistant strains, including meropenem-resistant and ceftazidime-non-susceptible (CAZ-NS) pathogens. Our focus encompasses various mechanisms of resistance, such as ESBL production, AmpC production, carbapenemase production, and other mechanisms like reduced porin expression. It is important to recognize that the use of CAZ-AVI should not be based solely on CAZ-NS status, and we will consider these diverse resistance mechanisms to assess CAZ-AVI’s role in managing challenging Gram-negative infections.

In this context, it is crucial to evaluate the clinical and microbiological outcomes associated with CAZ-AVI treatment compared to established antibiotic therapies. Understanding its efficacy against resistant strains can help guide healthcare professionals in making informed treatment decisions. Furthermore, the incorporation of CAZ-AVI into clinical practice highlights its potential role in antibiotic stewardship, a vital strategy aimed at optimizing antibiotic use to combat the growing issue of antimicrobial resistance.

This systematic review and meta-analysis will assess the efficacy of CAZ-AVI in treating Gram-negative infections, particularly those caused by CAZ-non-susceptible (NS) pathogens.

## Methods

### Search strategy

We conducted a systematic search of PubMed/MEDLINE, Embase, and Cochrane CENTRAL for clinical trials investigating Ceftazidime-Avibactam from January 1, 2013, to September 15, 2024. This systematic review adhered to the PRISMA guidelines for design and reporting (PROSPERO ID: CRD42024547247).

The search terms used were “Ceftazidime-Avibactam,” “Zavicefta,” “Ceftazidime plus Avibactam,” “Ceftazidime/Avibactam,” “CAZ-AVI,” “Avycaz,” “drug-resistant bacteria,” “antibiotic-resistant bacteria,” " drug-resistant Enterobacteriaceae,” “Gram-negative bacteria,” and “multidrug-resistant bacteria”.

### Study selection criteria

The inclusion criteria for articles consisted of clinical trials, including randomized controlled trials (RCTs) and Phase I-IV studies, evaluating the efficacy and safety of CAZ-AVI for Gram-negative bacterial infections. All retrieved records were consolidated, and duplicates were removed using EndNote X8 (Thomson Reuters, Toronto, ON, Canada). Two reviewers (NKH and MN) independently screened each record to assess eligibility based on predefined criteria. Studies unrelated to the topic were excluded after reviewing titles and abstracts, followed by a full-text review. Any disagreements between the reviewers were resolved by the lead investigator (MJN). Studies were included if they met the following Population, Intervention, Comparator, Outcome (PICO) criteria:

- **Patients**: Both adult and pediatric patients with Gram-negative infections, including Enterobacteriaceae and Pseudomonas aeruginosa, were included. Particular focus was given to those with strains not susceptible to ceftazidime alone.
- **Intervention**: Ceftazidime-Avibactam alone or in combination with other agents (e.g. metronidazole, aztreonam).
- **Comparators**: Other antibiotics (e.g., meropenem, doripenem, cefepime).
- **Outcomes**: Primary outcomes were clinical and microbiological success at the test-of-cure (TOC) visit. Secondary outcomes included adverse events and serious adverse events.

### Data extraction

Two reviewers (NKH and MN) systematically extracted data using a predefined spreadsheet in Microsoft Excel. Any discrepancies were resolved by involving a third reviewer (MJN). The extracted data included the first author, publication year, study design, country, patient demographics (age, sample size), intervention details and duration, pathogens and their susceptibility status, outcome assessment methods, and both clinical and microbiological outcomes.

### Quality assessment

Two reviewers (NKH, MNG) evaluated the quality of the studies using distinct assessment tools, with a third reviewer (MJN) intervening in case of inconsistencies. The Cochrane tool was utilized for experimental studies. The Cochrane tool is based on various criteria, including the use of random sequence generation, concealment of allocation to conditions, blinding of participants and personnel, blinding of outcome assessors, completeness of outcome data and other factors, as well as considerations for selective reporting and other biases. Each study was categorized as at low risk of bias when there was no concern regarding bias, at high risk of bias when there was concern, or unclear risk of bias if information was absent.

### Data analysis

Statistical analyses were performed using Comprehensive Meta-Analysis software, version 3.0 (CMA). Odds ratios and 95% confidence intervals (CIs) were calculated for the proportion of patients achieving treatment outcomes. Depending on the heterogeneity of effect sizes, either a random-effects or fixed-effects model was applied. Between-study heterogeneity was assessed using Cochran’s Q test and the I² statistic. Publication bias was evaluated using Begg’s test, with a p-value < 0.05 indicating statistically significant publication bias.

## Results

**Fig. 1:**
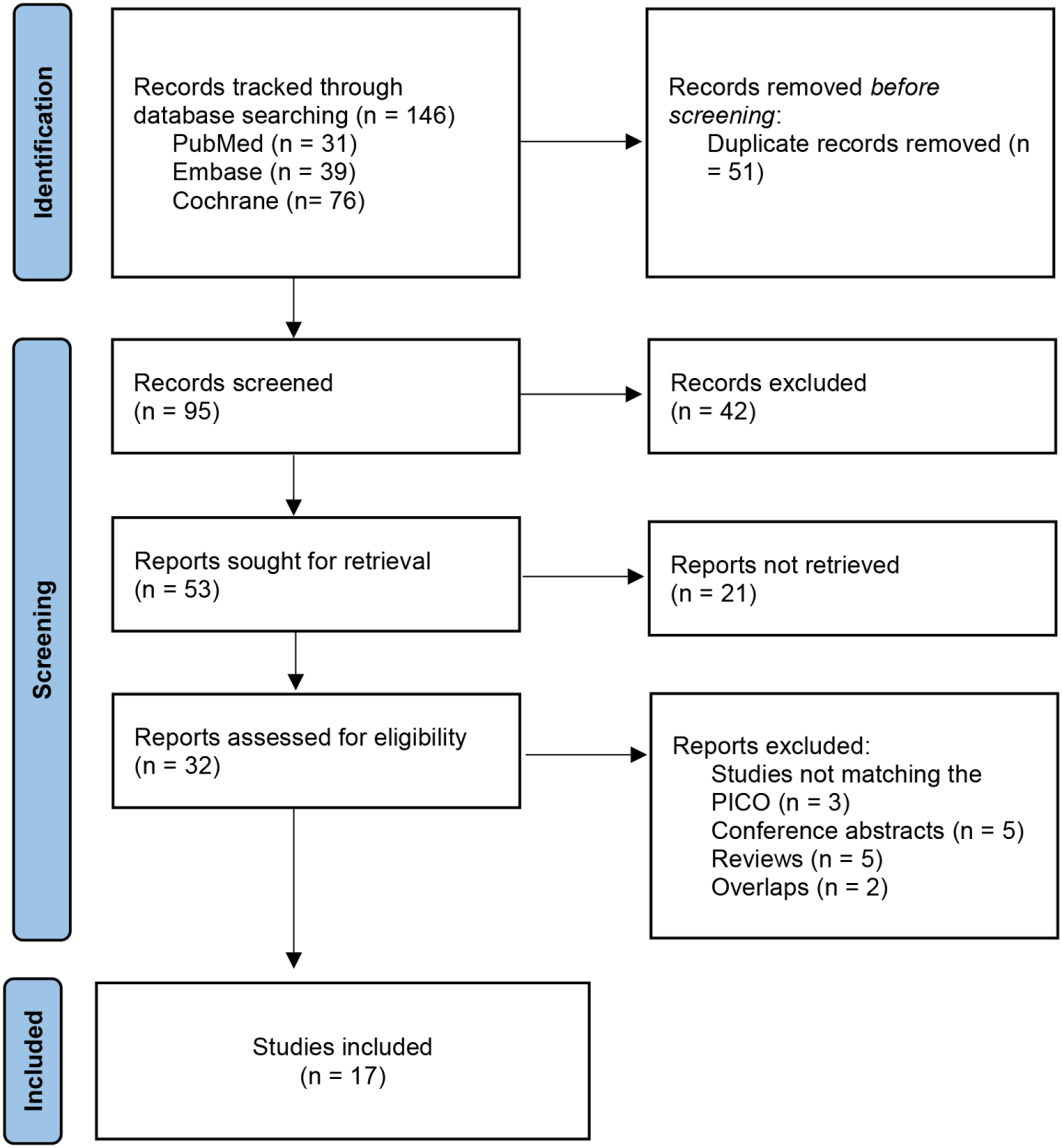
PRISMA flow diagram of study retrieval and eligibility

Using the outlined search strategy, 146 studies were identified through electronic database searches. After screening titles and abstracts, 32 full-text articles were retrieved for further evaluation. However, 15 studies were excluded for reasons detailed in Fig. 1. Ultimately, 17 studies were included: 9 RCTs to assess the efficacy of Ceftazidime-Avibactam, and the remaining Phase I to III trials for evaluating its adverse effects [5–21].

### Study characteristics

The included studies consisted of 17 clinical trials evaluating the efficacy and safety of CAZ-AVI against a range of Gram-negative infections (Table 1). These trials were conducted across diverse locations, including the USA, Belgium, the UK, and multiple Asian countries, and encompassed Phase 1, Phase 2, and Phase 3 designs. Sample sizes varied significantly, ranging from 16 to 1,043 participants, with age groups that included both pediatric (0–18 years) and adult populations (18–90 years), resulting in mean ages from 6.1 to 62.8 years.

**Table 1:** Basic information of the selected studies

Author	Year	Study place	Type of study	Sample size	Age range (years)	Mean age	Intervention	Control	Type of infection	Type of resistance	Priority Pathogens
Lodise et al. [5]	2022	USA	Phase 1	48	18–45	33.5	CAZ-AVI + Aztreonam	-	None	-	-
Bradley et al. [6]	2019	Multicenter	Phase 2	95	0–18	6.1	CAZ-AVI	Cefepime	UTI	CAZ-NS/ CAZ-S	Enterobacteriacae
Bradley et al. [7]	2019	Multicenter	Phase 2	83	0–18	10.3	CAZ-AVI + Metronidazole	Meropenem	IAI	CAZ-NS/ CAZ-S	Enterobacteriacae + *P. aeruginosa*
Torres et al. [8]	2019	Multicenter	Phase 3	870	18–90	62.8	CAZ-AVI	Meropenem	VAP	CAZ-NS/ CAZ-S	Enterobacteriacae + *P. aeruginosa*
Qin et al. [9]	2017	Asian Countries	Phase 3	431	18–90	48.5	CAZ-AVI + Metronidazole	Meropenem	IAI	CAZ-NS/ CAZ-S	Enterobacteriacae + *P. aeruginosa*
Torres et al. [10]	2017	Multicenter	Phase 3	726	18–90	62	CAZ-AVI	Meropenem	VAP	CAZ-NS/ CAZ-S	Enterobacteriacae + *P. aeruginosa*
Merdjan et al. [11]	2017	Belgium	Phase 1	31	18–90	48.1	CAZ-AVI	-	None	-	-
Carmeli et al. [12]	2016	Multicenter	Phase 3	302	18–90	62.6	CAZ-AVI	Carbapenem	IAI - UTI	CAZ-NS	Enterobacteriacae + *P. aeruginosa*
Mazuski et al. [13]	2016	Multicenter	Phase 3	1043	18–90	50	CAZ-AVI + Metronidazole	Meropenem	IAI	CAZ-NS/ CAZ-S	Enterobacteriacae + *P. aeruginosa*
Wagenlehner et al. [14]	2016	Multicenter	Phase 3	810	18–90	52.4	CAZ-AVI	Doripenem	IAI– UTI	CAZ-NS/ CAZ-S	Enterobacteriacae + *P. aeruginosa*
Bradley et al. [15]	2016	USA	Phase 1	32	0–18	9.1	CAZ-AVI	-	None	-	-
Li et al. [16]	2016	China	Phase 1	16	18–45	24	CAZ-AVI	-	None	-	-
Das et al. [17]	2015	UK	Phase 1	71	18–50	31.6	CAZ-AVI + Metronidazile	-	None	-	-
Merdjan et al. [18]	2015	Europe	Phase 1	111	18–45	29.8	CAZ-AVI	Avibactam	None		-
Tominaga et al. [19]	2015	USA (Japanese Patients)	Phase 1	16	20–45	28.8	CAZ-AVI	Avibactam	None		-
Das et al. [20]	2014	USA	Phase 1	51	18–45	26	CAZ-AVI	Avibactam	None	-	-
Lucasti et al. [21]	2013	Multicenter	Phase 2	203	18–90	42.8	CAZ- AVI + Metronidazole	Meropenem	IAI	CAZ-NS/ CAZ-S	Enterobacteriacae + *P. aeruginosa*

*CAZ-AVI: Ceftazidime-Avibactam*,* IAI: Intraabdominal infection*,* UTI: Urinary tract infection*,* VAP: Ventilator associated pneumonia*,* NM: Not mentioned*,* BAT: Best available therapy*

The primary intervention across studies was CAZ-AVI, which was administered either as a monotherapy or in combination with other antibiotics such as Aztreonam and Metronidazole. Comparators included cefepime, meropenem, doripenem, and placebo, depending on the specific study design. The trials addressed various types of infections, including urinary tract infections (UTIs), intra-abdominal infections (IAIs), and ventilator-associated pneumonia (VAP). A significant focus was placed on pathogens, particularly Enterobacteriaceae and *Pseudomonas aeruginosa*, with studies reporting on infections caused by both CAZ -susceptible and non-susceptible (NS) strains, highlighting the urgency of addressing antimicrobial resistance in these priority pathogens.

CAZ-NS refers to bacterial strains with reduced susceptibility to the antibiotic CAZ, as determined by antimicrobial susceptibility testing, indicating that these bacteria do not respond effectively to this treatment.

### Quality assessment

Quality assessment was conducted exclusively for the studies included in the meta-analysis. Among the total of 9 studies, 6 articles demonstrated a low risk of bias across all categories. However, 3 studies exhibited some risk of bias specifically in the blinding of participants and personnel category. Additionally, 2 studies were found to have a high risk of bias in the blinding of outcome assessors category (Table 2).

**Table 2:** Quality assessment of the studies included in the meta-analysis (the Cochrane tool)

Author	Random sequence generation	Allocation concealment	Blinding of participants and personnel	Blinding of outcome assessment	Incomplete outcome data	Selective reporting	Other bias
Bradley, Roilides	Low risk	Low risk	High risk	Low risk	Low risk	Low risk	Low risk
Bradley, Broadhurst	Low risk	Low risk	High risk	Low risk	Low risk	Low risk	Low risk
Carmeli	Low risk	Low risk	High risk	High risk	Low risk	Low risk	Low risk
Lucasti	Low risk	Low risk	Low risk	Low risk	Low risk	Low risk	Low risk
Mazuski	Low risk	Low risk	Low risk	Low risk	Low risk	Low risk	Low risk
Qin	Low risk	Low risk	Low risk	Low risk	Low risk	Low risk	Low risk
Torres2017	Low risk	Low risk	Low risk	Low risk	Low risk	Low risk	Low risk
Torres2019	Low risk	Low risk	Low risk	Low risk	Low risk	Low risk	Low risk
Wagenlehner	Low risk	Low risk	Low risk	Low risk	Low risk	Low risk	Low risk

### Efficacy of CAZ-AVI against Gram-negative pathogens

**Fig. 2:**
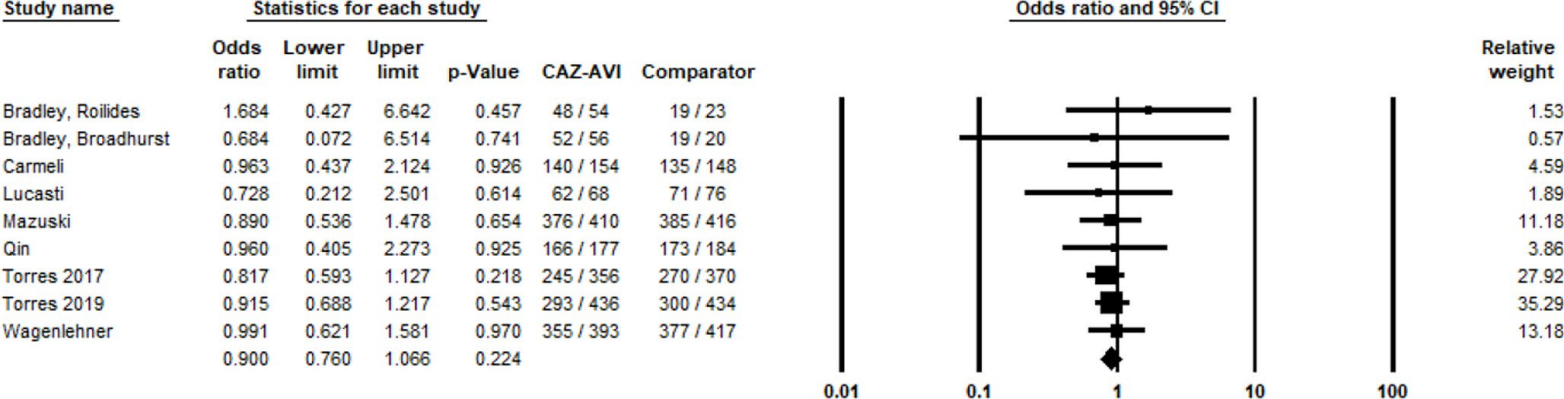
Clinical success of CAZ-AVI against Gram-negative pathogens

**Fig. 3:**
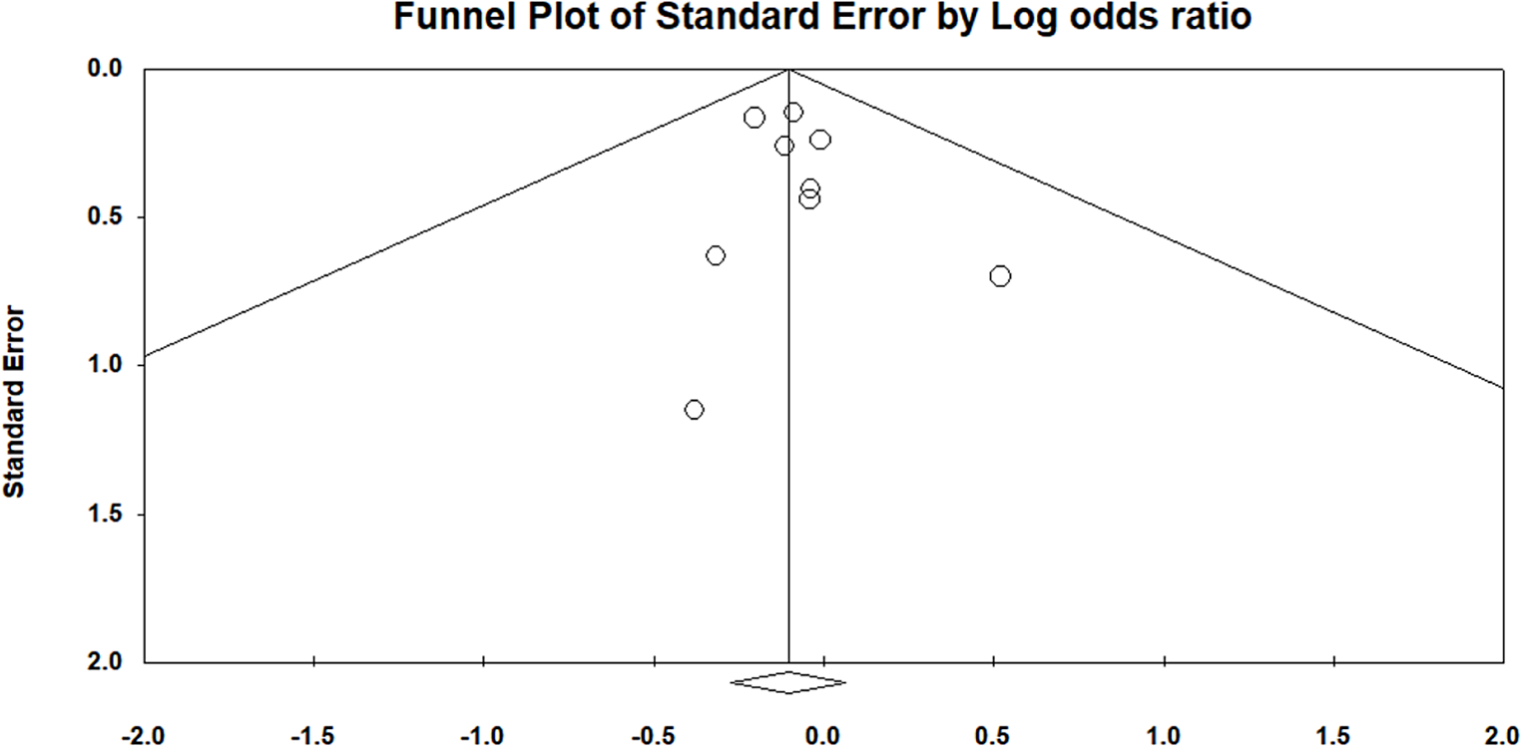
Funnel plot assessing the studies included in clinical assessment

A total of 9 studies reported data on the clinical success of CAZ-AVI in treating Gram-negative infections compared to other antibiotic treatments. The meta-analysis revealed a non-significant difference in clinical cure rates between CAZ-AVI and the comparators, which included antibiotics such as meropenem and cefepime, with a pooled OR of 0.90 (95% CI: 0.76 to 1.07) and a p-value of 0.22 (Fig. 2). There was no evidence of publication bias, as indicated by a p-value greater than 0.05 (Fig. 3).

**Fig. 4:**
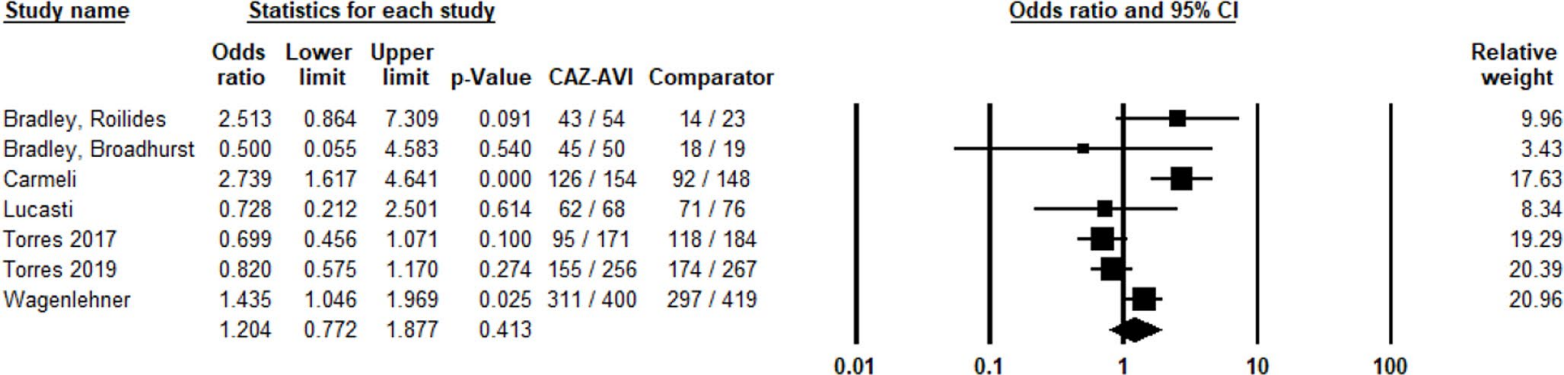
Microbiological success of CAZ-AVI against Gram-negative pathogens

**Fig. 5:**
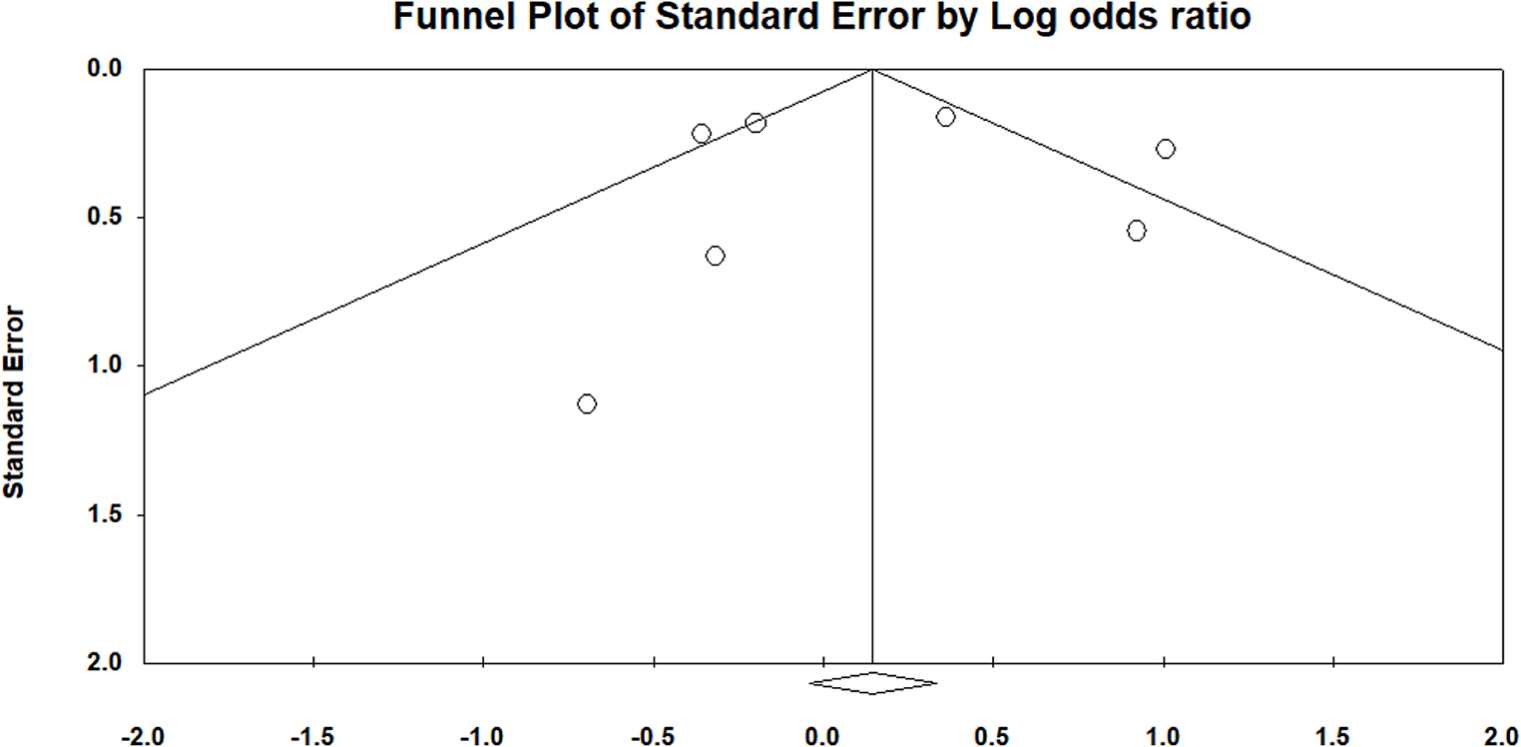
Funnel plot assessing the studies included in microbiological assessment

Additionally, 7 studies provided data on the microbiological success of CAZ-AVI relative to the comparators. The meta-analysis indicated a non-significant increase in microbiological cure rates for CAZ-AVI compared to these treatments, with a pooled OR of 1.20 (95% CI: 0.77 to 1.88) and a p-value of 0.41 (Fig. 4). Similar to the clinical success analysis, there was no evidence of publication bias (p-value > 0.05) (Fig. 5).

### Efficacy of CAZ-AVI against Gram-negative CAZ-NS pathogens

**Fig. 6:**
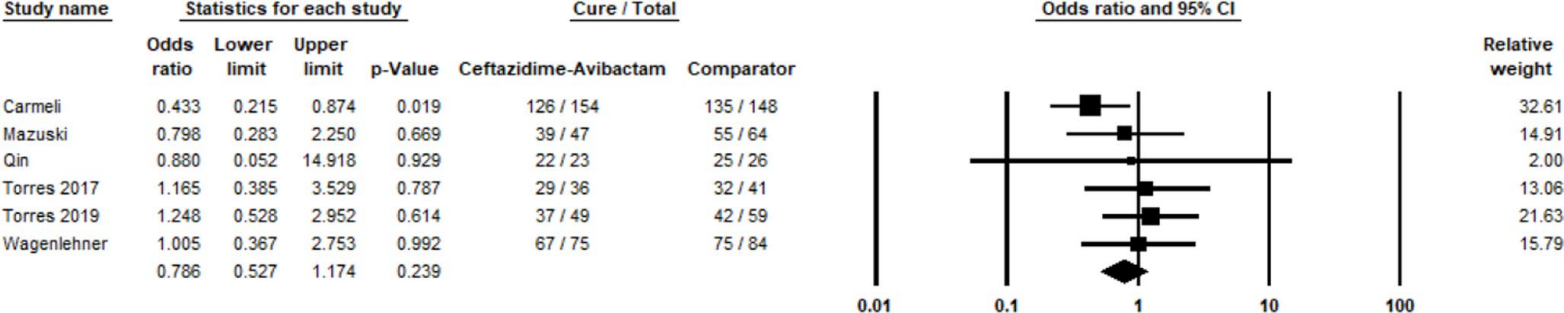
Clinical success of CAZ-AVI against Gram-negative CAZ-NS pathogens

Six studies assessed the clinical success of CAZ-AVI compared to other treatments for CAZ-NS pathogens. The meta-analysis showed no significant difference in clinical cure rates, with a pooled OR of 0.77 (95% CI: 0.53 to 1.17) and a p-value of 0.24 (Fig. 6). No publication bias was found (p-value > 0.05).

**Fig. 7:**
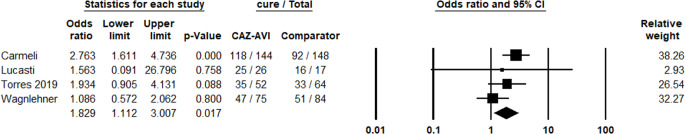
Microbiological success of CAZ-AVI against Gram-negative CAZ-NS pathogens

Additionally, four studies reported microbiological success. The meta-analysis indicated a significant increase in microbiological cure rates for CAZ-AVI, with a pooled OR of 1.83 (95% CI: 1.11 to 3.00) and a p-value of < 0.02 (Fig. 7), with no evidence of publication bias (p-value > 0.05).

### Subgroup analysis

Data from two studies focused on children, while seven studies assessed adults. In children, the pooled OR for clinical cure rates was 1.32 (95% CI: 0.41 to 4.26, *p* = 0.64), and for microbiological cure rates, it was 1.50 (95% CI: 0.35 to 6.57, *p* = 0.58). Publication bias could not be assessed due to the limited number of studies.

In adults, the pooled OR for clinical cure rates from seven studies was 0.89 (95% CI: 0.75 to 1.06, *p* = 0.20), with no evidence of publication bias (*p* > 0.05). For microbiological cure rates assessed in five studies, the pooled OR was 1.15 (95% CI: 0.71 to 1.86, *p* = 0.58), again showing no publication bias (*p* > 0.05).

### Adverse effects

For assessing adverse effects of CAZ-AVI, we used information provided on the matter from 17 studies. Adverse effects were mostly related to GI disorders such as diarrhea, constipation, vomiting and nausea which were present in most of the studies we assessed, diarrhea being the most common. Other adverse effects included respiratory disorders, cardiac disorders, nervous system disorders, skin and subcutaneous disorders, pyrexia, etc. The most common adverse effects on studies are available in Table 3.

**Table 3:** Adverse effects

Author	No. of patients	Any AEs	SAEs and AEs of severe intensity	Deaths	Discontinuation due to AEs	GI disorders	Respiratory disorders	Skin and subcutaneous disorders	Nervous System disorders	Cardiac disorders	Anemia	Nausea	Diarrhea	Constipation	Vomiting	Abdominal pain	Headache	Hypertension	Hypotension	Cough	Pleural effusion	Peripheral edema	Pyrexia
Lodise et al. [5]	48	46	6	0	8	NM	NM	NM	NM	21*	NM	NM	NM	NM	NM	NM	7	NM	NM	NM	NM	NM	NM
Bradley et al. [6]	67	36	8	0	3	9	3	7	NM	NM	NM	2	5	1	2	2	NM	NM	NM	2	NM	NM	2
Bradley et al. [7]	61	32	5	0	0	13	2	NM	0	NM	0	NM	NM	NM	9	0	NM	NM	NM	1	NM	NM	2
Torres et al. 8]	436	323	147	26	16	131	30	19	NM	15	25	14	67	25	25	NM	NM	14	10	NM	10	17	14
Qin et al. [9]	215	NM	14	0	7	41	13	NM	NM	NM	NM	18	13	5	5	NM	3	NM	NM	8	NM	NM	9
Torres et al. [10]	405	302	141	25	16	122	29	17	NM	21	25	13	61	25	23	10	NM	14	10	NM	9	17	10
Merdjan et al. [11]	31	19**	0	0	0	NM	NM	NM	NM	NM	NM	NM	NM	NM	NM	NM	NM	NM	NM	NM	NM	NM	NM
Carmeli et al.[12] (UTI)	152	43	NM	7	1	21	NM	NM	NM	1	NM	5	3	NM	4	3	1	NM	NM	NM	NM	3	4
Carmeli et al. (IAI)	12	8	NM				NM	NM	NM	NM	NM	3	2	NM	2	0	2	NM	NM	NM	NM	0	0
Mazuski et al. [13]	529	243	72	13	14	118	11	NM	15	NM	11	36	40	8	24	NM	15	15	12	11	NM	NM	24
Wagenlehner et al. [14]	511	185	31	0	7	40	NM	NM	38	NM	NM	15	14	11	NM	NM	38	NM	NM	NM	NM	NM	NM
Bradley et al. [15]	32	6	0	0	NM	3	NM	NM	NM	1	NM	0	1	1	1	NM	NM	NM	NM	NM	NM	NM	NM
Li et al. [16]	12	3	0	0	0	0	0	0	0	0	0	0	0	0	0	0	0	0	0	0	0	0	0
Das et al. [17]	28	22	0	0	NM	16	NM	14	NM	NM	NM	2	4	NM	NM	2	4	NM	NM	NM	NM	NM	NM
Merdjan et al. [18]	16	4	0	0	0	0	0	0	0	0	0	0	0	0	0	0	0	0	0	0	0	0	0
Tominaga et al. (Single dose study)	7	0	0	0	0	0	0	0	0	0	0	0	0	0	0	0	0	0	0	0	0	0	0
Tominaga et al.(Multiple dose study)		1																	1				
Das et al. [20]	46	14	0	0	1	NM	NM	NM	NM	NM	NM	1	NM	NM	1	NM	1	NM	NM	NM	NM	NM	NM
Lucasti et al.	101	65	1	3	5	32	6	NM	NM	1	NM	10	NM	NM	14	8	NM	NM	NM	6	NM	NM	9

AE: adverse effect, GI: gastrointestinal, NM: not mentioned

*Cardiac disorders included 16 cases of bradycardia and 5 cases of prolonged QT, the numbers were summed

**The AEs occurred in 9 subjects in renally impaired group

## Discussion

The analysis of CAZ-AVI’s efficacy against Gram-negative pathogens revealed comparable clinical success rates to comparative treatments. For patients with CAZ-NS pathogens, clinical success rates were similar; however, CAZ-AVI demonstrated a non-significant potential increase in microbiological cure rates. These findings indicate that although clinical outcomes may not significantly differ, CAZ-AVI may still provide some benefits in microbiological success, particularly against CAZ-NS pathogens.

### Clinical implications

The findings underscore the importance of incorporating CAZ-AVI into treatment guidelines for Gram-negative infections, especially those involving CAZ-Non-Susceptible pathogens. This strategy can enhance the management of resistant infections, improve patient outcomes, and potentially reduce healthcare costs associated with prolonged illness [22–24]. Policymakers should prioritize the availability and accessibility of CAZ-AVI in clinical settings, while physicians should view it as a viable treatment option for patients facing resistance challenges [25].

Dosing considerations are important for patients with pre-existing renal impairment, as CAZ-AVI can potentially worsen renal function [26]. Although CAZ-AVI is typically used when treatment options are limited and most reported adverse effects are mild to moderate in severity [27], its potential adverse effects should still be taken seriously.

Furthermore, the role of antibiotic stewardship programs is crucial in combating antibiotic resistance [28–30]. By promoting the responsible use of CAZ-AVI, healthcare providers can mitigate the emergence of further resistance. Educating clinicians on the appropriate selection and dosing of CAZ-AVI is essential for maximizing efficacy while minimizing adverse effects and resistance development [31, 32]. Implementing strict infection control measures, such as hand hygiene and environmental cleaning, will also help reduce the spread of resistant pathogens [33–35]. Additionally, regular monitoring of resistance patterns and patient outcomes should be integrated into stewardship initiatives to ensure judicious use of CAZ-AVI [36]. This collaborative effort will contribute to more sustainable antibiotic use and better management of resistant infections within the healthcare system.

### Study limitations

This study has several limitations. Firstly, the included studies varied in design, sample size, and patient demographics, which may affect the generalizability of the findings. The heterogeneity of the studies could influence the pooled estimates and complicate result interpretation.

Secondly, the limited number of studies assessing clinical and microbiological success rates for CAZ-AVI against CAZ-Non-Susceptible pathogens restricts the robustness of the conclusions. Additionally, publication bias could not be fully assessed in some analyses due to the small number of studies.

Moreover, reliance on available data means some relevant studies may not have been included, potentially affecting the overall analysis. Differences in definitions of clinical and microbiological success among studies may also introduce variability in reported outcomes. Lastly, while this study highlights the importance of antibiotic stewardship, further research is needed to evaluate the long-term outcomes and resistance patterns associated with CAZ-AVI use.

## Conclusions

This study suggests that CAZ-AVI is an effective option for treating Gram-negative infections, particularly those caused by CAZ-NS pathogens. While overall clinical and microbiological success rates are comparable to other antibiotics, CAZ-AVI demonstrates notable advantages in managing resistant infections, especially in achieving significantly better microbiological success rates against CAZ-NS pathogens. The findings emphasize the importance of incorporating CAZ-AVI into treatment guidelines and highlight the critical role of antibiotic stewardship programs to optimize its use and mitigate resistance. Ongoing monitoring of resistance patterns and patient outcomes is crucial for ensuring effective treatment strategies and enhancing patient care.

## Data Availability

No datasets were generated or analysed during the current study.
